# Exosomes secreted by endothelial progenitor cells accelerate bone regeneration during distraction osteogenesis by stimulating angiogenesis

**DOI:** 10.1186/s13287-018-1115-7

**Published:** 2019-01-11

**Authors:** Yachao Jia, Yu Zhu, Shuo Qiu, Jia Xu, Yimin Chai

**Affiliations:** Department of Orthopedic Surgery, Shanghai Jiao Tong University Affiliated Sixth People’s Hospital, Yishan Rd 600, Shanghai, 200233 People’s Republic of China

**Keywords:** Distraction osteogenesis, Exosomes, Endothelial progenitor cells, Bone regeneration, Angiogenesis

## Abstract

**Background:**

Distraction osteogenesis (DO) is an effective but lengthy procedure to fully induce bone regeneration in large bone defects. Accumulating evidence supports the role of exosomes secreted by endothelial progenitor cells (EPC-Exos) in stimulating angiogenesis, which is closely coupled with osteogenesis. This study aimed to investigate whether EPC-Exos promote bone regeneration during DO in rats.

**Methods:**

Exosomes were isolated from the supernatants of rat bone marrow EPCs via ultracentrifugation and characterized via transmission electron microscopy, tunable resistive pulse sensing analysis, and western blot analysis. Unilateral tibial DO models were generated using 68 Sprague-Dawley rats with a distraction rate of 0.5 mm per day for 10 days. After local injection of EPC-Exos into the distraction gaps after distraction, the therapeutic effects of EPC-Exos on bone regeneration and angiogenesis were assessed via X-ray, micro-computed tomography (micro-CT), and biomechanical and histological analyses. Pro-angiogenic effects and the potential mechanism underlying the effects of EPC-Exos on human umbilical vein endothelial cells were subsequently evaluated via in vitro assays including Cell Counting Kit-8, wound healing, tube formation, and western blot assays.

**Results:**

EPC-Exos were spherical or cup-shaped vesicles ranging from 50 to 150 nm in diameter and expressed markers including CD9, Alix, and TSG101. X-ray, micro-CT, and histological analyses revealed that bone regeneration was markedly accelerated in rats treated with EPC-Exos. The distracted tibias from the Exos group also displayed enhanced mechanical properties. Moreover, vessel density was higher in the Exos group than in the control group. In addition, in vitro analyses revealed that EPC-Exos enhanced the proliferation, migration, and angiogenic capacity of endothelial cells in an miR-126-dependent manner. Further, EPC-Exos downregulated SPRED1 and activated Raf/ERK signaling.

**Conclusions:**

The present results show that EPC-Exos accelerate bone regeneration during DO by stimulating angiogenesis, suggesting their use as a novel method to shorten the treatment duration of DO.

## Background

Distraction osteogenesis (DO) is the first-line treatment method for long bone defects due to trauma or surgical resection, especially in cases of postsurgical complications and infections [1–3]. The DO procedure comprises three phases: the latency phase after osteotomy and application of external fixators, the distraction phase wherein the bone segments proximal and distal to the osteotomy site are separated via gradual and continuous distraction, and the consolidation phase for neo-osteogenesis and consolidation until achievement of sufficient quality [4]. Despite the unique ability to fully induce neo-osteogenesis, this lengthy technique is limited by the undesired long duration of the consolidation phase and a subsequent increase in the risk of complications [5]. Therefore, accelerating callus formation and consolidation during DO and shortening the external fixation time are of great clinical significance.

Osteogenesis is reportedly closely coupled with angiogenesis during neo-osteogenesis [6]. Robust angiogenesis has been identified during DO [7, 8]. Moreover, impairment of angiogenesis by radiation or age reportedly inhibits bone regeneration in DO [9–11]. Furthermore, stimulation of angiogenesis with various cytokines has yielded promising results during DO in animal models [12, 13]. However, clinical application of cytokines is generally limited owing to rapid clearance, high cost, potential toxicity, or uncertain effects. Transplantation of endothelial progenitor cells (EPCs), the precursor of endothelial cells, reportedly stimulated angiogenesis by differentiating into mature endothelial cells or triggering angiogenic events by secreting various trophic factors [14–17]. These properties make EPCs an attractive candidate for stem cell therapy. However, EPC transplantation has some limitations, including emboli formation, immunogenicity, and malignant transformation [18].

Recently, accumulating evidence indicates that stem cells may exert their therapeutic effects in tissue repair and regeneration through exosomes secretion [19–21]. Exosomes are lipid bilayer membrane-bound vesicles with a diameter from 30 to 150 nm [22]. These vesicles are released by almost all cell types and contain various bioactive proteins, lipids, and RNAs [23]. After being endocytosed by recipient cells, exosomes can regulate target cell function by transferring RNAs and proteins. When compared with stem cell therapy, application of exosomes without DNA and HLA antigens can prevent the risk of tumorigenicity and immunogenicity. Previous studies have reported that exosomes secreted by EPCs (EPC-Exos) promote angiogenesis in diabetic wound healing and accelerate re-endothelialization after vascular injury [24, 25]. However, it remains unknown how EPC-Exos influence the process of DO.

Hence, the present study aimed to evaluate the therapeutic potential of EPC-Exos on osteogenesis and consolidation during DO in rats.

## Methods

### Animals

All animal experimental protocols in this study were reviewed and approved by the Institute of Animal Care and Use Committee of Shanghai Jiao Tong University Affiliated Sixth People’s Hospital. Sprague-Dawley rats were used in this study and fed with standard laboratory water and food under conventional conditions.

### Cell culture

Bone marrow EPCs were harvested from 4-week-old Sprague-Dawley rats as described previously [26]. Briefly, the bone marrow cells were harvested, overlaid on Histopaque (1.083 g/ml, Sigma-Aldrich, St. Louis, MO), and centrifuged at 400×*g* for 30 min at room temperature. Thereafter, mononuclear cells were harvested, washed with phosphate-buffered saline (PBS), plated in culture dishes pre-coated with rat fibronectin (Merck, Darmstadt, Germany), and cultured in endothelial basal medium 2 (EBM-2; Lonza, Basel, Switzerland) supplemented with EGM-2 MV SingleQuots. Non-adherent cells were eliminated after 3 days, and media were replaced every alternate day. Cells were cultured at 37 °C, 5% CO_2_ in a humidified environment and passaged at 80–90% confluence. EPCs at passages 2 to 4 were used in the following experiments. EPCs were transfected with miR-126-3p inhibitor or scrambled control (RiboBio, Guangzhou, China) at a concentration of 50 nM and Lipofectamine® 2000 transfection reagent (Gibco Life Technologies) according to the manufacturer’s instructions.

Human umbilical vein endothelial cells (HUVECs) were obtained from Sciencell Research Laboratories (San Diego, CA, USA) and cultured in medium 200 (M200; Gibco, Carlsbad, CA, USA) supplemented with 2% low serum growth supplement (Cascade Biologics, Portland, OR, USA).

### Isolation and purification of EPC-Exos

EPC-Exos were isolated from the supernatants of EPCs as described previously [25]. Briefly, after approaching approximately 80% confluence, EPCs were washed twice with PBS and cultured in media containing exosome-free fetal bovine serum for an additional 48 h. The supernatants were harvested, centrifuged (300×*g* for 10 min and 2000×*g* for another 10 min), and filtered through a 0.22-μm filter (Millipore, Billerica, MA, USA) to eliminate cells and cellular debris. Filtered supernatants were then transferred to Amicon Ultra-15 centrifugal filter devices (Millipore) and centrifuged at 4000×*g* to approximately 200 μl. The ultrafiltrate was washed with PBS and re-ultrafiltered to 200 μl. For purification of exosomes, the ultrafiltrate was laid onto a 30% sucrose-D_2_O cushion in a sterile Ultra-Clear™ tube (Beckman Coulter, Kraemer Boulevard Brea) and ultracentrifuged at 100,000×*g* for 70 min (Sorvall, Avanti J-26XP, fixed angle rotor; Beckman Coulter). The exosomal pellet was suspended in filtered PBS. All centrifugation procedures were performed at 4 °C. EPCs-Exos were used for subsequent experiments or stored in aliquots at − 80 °C. Exos derived from EPCs and EPCs transfected with miR-126-3p inhibitor or scrambled control were defined as Exos, 126i-Exos, and NC-Exos.

### Characterization of EPCs-Exos

Exosome morphology was assessed via transmission electron microscopy (TEM; FEI, Eindhoven, Netherlands) after negative staining with 2% uranyl acetate for 30 s. The size distribution and density of EPC-Exos were measured using tunable resistive pulse sensing (TRPS) analysis by qNano (Izon Science, Cambridge, MA, USA) as described previously [25]. The characteristic proteins of exosomes including CD9, Alix, TSG101, and negative marker Calnexin (1:1000; Abcam, Cambridge, UK) were analyzed via western blot analysis [27].

### Animal surgery

Sixty-eight adult male Sprague-Dawley rats were used to generate the tibial DO model and then assigned to the control, Exo-1 group, Exo-2, or EPCs groups (*n* = 17 per group). The tibial DO procedures were carried out as previously described [28]. Briefly, a transverse osteotomy was performed at the midshaft of the right tibia after anesthesia and exposure. Thereafter, a monolateral external fixator (Xinzhong Company, Tianjin, China) was mounted to fix the proximal and distal segments of the tibia. Thereafter, surgical incisions were closed layer wise. The DO procedures comprise three phases: the latency phase for 5 days, the distraction phase for 10 days (0.25 mm every 12 h), and the consolidation phase for 4 weeks. At the beginning of the consolidation phase, all rats received a local injection of PBS, 1 × 10^10^ EPC-Exos (Exo-1), 1 × 10^11^ EPC-Exos (Exo-2), or 1 × 10^6^ EPCs (EPCs) into the distraction gaps. EPC-Exos and EPCs were suspended in 100 μl PBS. Four additional rats without osteotomy were used as the sham control. The tibia specimens were harvested 2 (*n* = 5 per group) and 4 weeks (*n* = 12 per group) after distraction.

### Exosome distribution after injection

To monitor the in vivo distribution of exosomes after local injection, one rat from each group was treated with DiR-labeled (Life Technologies, Carlsbad, CA, USA) exosomes or EPCs and imaged 2 weeks after injection using the IVIS Spectrum Imaging System (PerkinElmer, USA) [29].

### Digital radiography and micro-computed tomography

X-ray imaging of the distraction gap was performed 2 and 4 weeks after distraction. Micro-computed tomography (Micro-CT; SKYSCAN 1176, Bruker, Kontich, Belgium) was performed to quantify the regenerated bone in the distraction zone. Thereafter, three-dimensional (3D) reconstructions of the regenerated callus were obtained using the CTVol software (Skyscan Company). Furthermore, parameters of the regenerated bone, including bone volume/tissue volume (BV/TV) and bone mineral density (BMD), were analyzed using the CTAn software (Skyscan Company).

### Perfusion protocol

To evaluate angiogenesis in the distraction regenerates, rats were perfused using Microfil (Microfil MV-122; Flow Tech Inc.; Carver, MA, USA) at 4 weeks after distraction as described previously [30]. Briefly, after anesthesia with 3% sodium pentobarbital (50 mg/kg), the thoracic cavity was opened and left ventricular catheterization was performed using an infusion needle. Thereafter, heparinized normal saline, 10% normal buffered formalin, and 10 ml mixed Microfil solution were perfused. The perfused rats were stored at 4 °C overnight to ensure polymerization of the contrast agent. Thereafter, the tibia samples were harvested, demineralized, and subjected to micro-CT.

### Three-point bending mechanical analysis

The mechanical strength of the distracted tibia samples was assessed using a three-point bending device (Instron5566; Instron, Norwood, MA, USA). The tibia samples were loaded in the anterior-posterior direction at a loading rate of 1 mm/min until failure. Maximum loading and energy to failure were analyzed and normalized to the contralateral tibia.

### Histological analysis

After fixation in 10% neutral formalin for 48 h, the tibia specimens were decalcified in 10% EDTA for 4 weeks and embedded in paraffin. Thin sections (5-μm-thick) were cut along the longitudinal axis of each specimen along the sagittal plane for hematoxylin-eosin (HE) and Masson’s trichrome staining. Immunohistochemistry staining was performed using primary antibodies to rabbit CD31 (Abcam, 1:100) and osteocalcin (OCN; Santa Cruz, 1:100) overnight at 4 °C. Then, a horseradish peroxidase-streptavidin detection system (Dako, USA) was used, followed by counterstaining with hematoxylin.

### Assessment of in vitro effects of EPC-Exos on HUVECs

The effects of EPC-Exos on the proliferation, migration, and angiogenic ability of HUVECs were also assessed to evaluate the pro-angiogenic capacity of EPC-Exos.

Cell proliferation was assessed using the Cell Counting Kit-8 (CCK-8; Dojindo, Kyushu Island, Japan) assay. HUVECs (1 × 10^4^ per well) were seeded in 96-well plates with medium containing PBS, Exo-1 (1 × 10^10^/ml), Exo-2 (1 × 10^11^/ml), NC-Exo (1 × 10^11^/ml), or 126i-Exo (1 × 10^11^/ml). After co-culture for 1, 3, and 5 days, 100 μl of fresh complete medium and 10 μl CCK-8 solution were added to each well, and cells were incubated for another 2 h. Thereafter, the optical density was measured at 450 nm, using a microplate reader.

For the wound healing assay, HUVECs (2 × 10^5^ cells per well) were plated in 12-well plates and incubated at 37 °C until confluence. Thereafter, the confluent monolayer was scratched using a pipette tip, washed with PBS to eliminate cell debris, and cultured in media supplemented with PBS, Exo-1 (1 × 10^10^/ml), Exo-2 (1 × 10^11^/ml), NC-Exo, or 126i-Exo. Cells were photographed at 0 and 10 h after scratching. The wound closure rate was determined as reported previously [27].

For the tube formation assay, HUVECs (1 × 10^4^ cells per well) were seeded in 96-well plates coated with growth factor-reduced Matrigel (BD Biosciences, Franklin Lakes, NJ, USA) and incubated in media containing PBS, Exo-1 (1 × 10^10^/ml), Exo-2 (1 × 10^11^/ml), NC-Exo, or 126i-Exo. Tube formation was examined after 6 h, using an inverted microscope (Leica, Germany). Total tube length per image and branch points were measured using ImageJ software (National Institutes of Health, Bethesda, MD, USA).

### Western blot analysis

The effects of EPC-Exos on the levels of hypoxia-inducible factor-1α (HIF-1α), SPRED1, and activation of Ras/ERK signaling were detected via western blot analysis. Western blot analysis was performed as described previously [31]. The primary antibodies used were anti-SPRED1, anti-Raf, phosphorylated Raf (p-Raf), anti-Erk1/2, anti-pErk1/2, and anti-HIF-1α (Cell Signaling Technology).

### Quantitative reverse transcription polymerase chain reaction analysis

Total RNA was extracted using Trizol reagent (Invitrogen, Carlsbad, CA, USA). Complementary DNA was prepared using Superscript III Reverse Transcriptase (Life Technologies, Carlsbad, CA, USA). Quantitative PCR for vascular endothelial growth factor-A (VEGFA), basic fibroblast growth factor (bFGF), transforming growth factor beta 1 (TGFB1), angiogenin (ANG), platelet-derived growth factor (PDGF), and miR-126 was performed using the SYBR-Green Master Mix Plus (Toyobo, Osaka, Japan) with an ABI 7900HT System (Applied Biosystems, Foster City, CA, USA). The primers were purchased from Guangzhou RiboBio Co., Ltd. (Guangzhou, China) (Table 1). The results were normalized using GAPDH for mRNA or U6 for miR-126.

**Table 1:** Primers used in this study for qRT-PCR

Genes	Forward primer (5′-3′)	Reverse primer (5′-3′)
h-VEGFA	CGCTCGGTGCTGGAATTTGA	AGTGGGGAATGGCAAGCAAA
h-bFGF	CAATTCCCATGTGCTGTGAC	ACCTTGACCTCTCAGCCTCA
h-TGFB1	TTGAGGGCTTTCGCCTTAGC	TGAACCCTGCGTTGATGTCC
h-ANG	ACACTTCCTGACCCAGCACT	TGTTTTCACAGATGGCCTTG
h-GAPDH	ATCCCATCACCATCTTCC	GAGTCCTTCCACGATACCA

### Statistical analysis

All data are presented as mean ± standard deviation values. Differences in study variables were analyzed using Student’s *t* test for two groups or one-way ANOVA followed by Turkey’s post hoc test for three or more groups, using GraphPad Prism 5, and *P* < 0.05 was considered statistically significant.

## Results

### Characterization of EPC-Exos

**Fig. 1:**
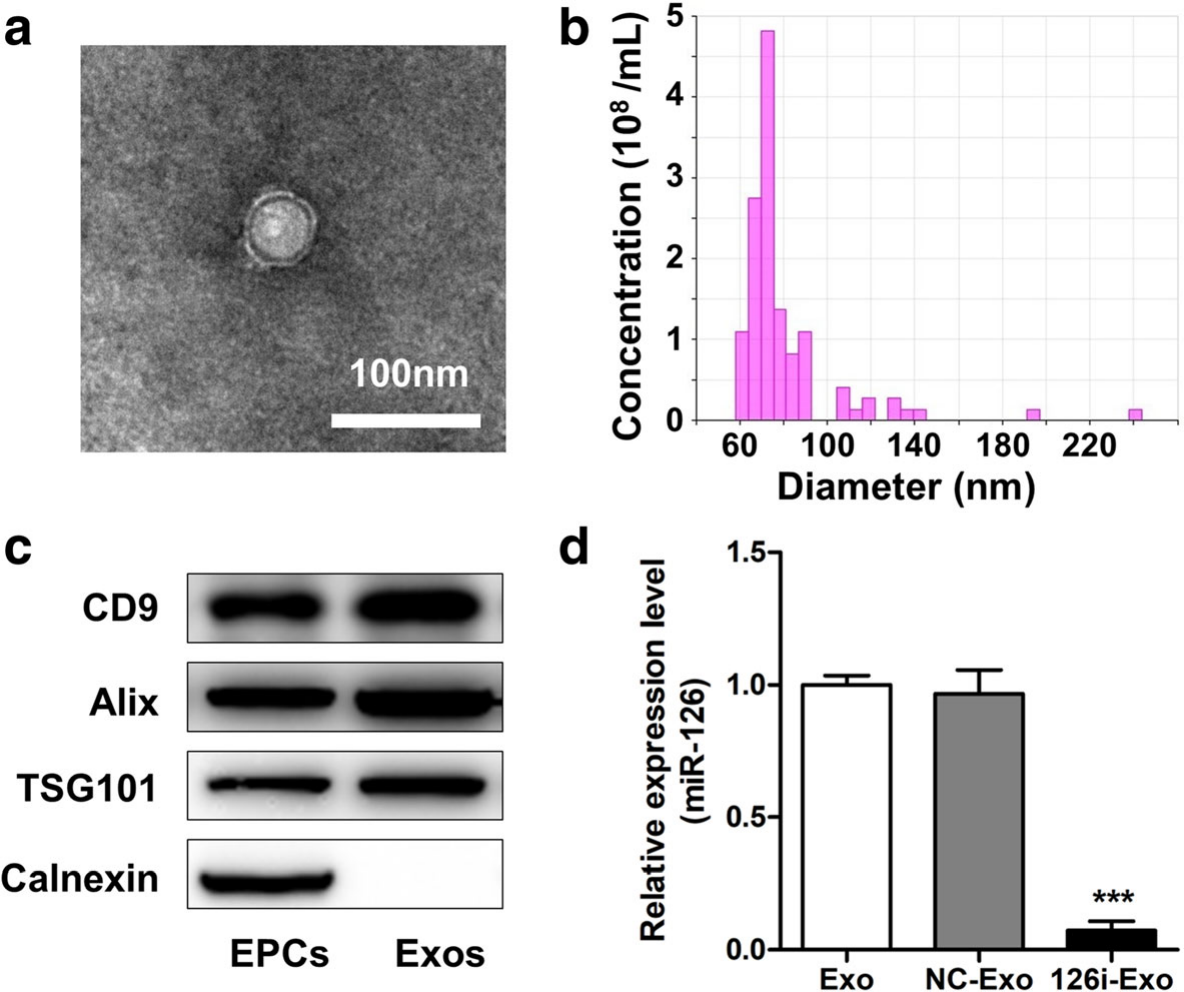
Characterization of exosomes secreted by rat endothelial progenitor cells (EPC-Exos). **a** Morphology of EPC-Exos by transmission electron microscopy. **b** Particle size distribution of EPC-Exos measured by tunable resistive pulse sensing analysis. **c** Western blot analysis of exosomal marker proteins (CD9, Alix, TSG101), and negative marker (Calnexin) in EPC-Exos. **d** EPCs were transfected with miR-control and anti-miR-126 to produce NC-Exos and 126i-Exos. The relative expression level of miR-126 was measured by qRT-PCR. ***P < 0.001 vs Exos and NC-Exos. *EPCs* endothelial progenitor cells, *Exo* exosomes secreted by EPCs

A representative TEM image of EPC-Exos was shown in Fig. 1a. The EPC-Exos were cup- or round-shaped vesicles. TPRS analyses demonstrated that the majority of EPC-Exos were approximately 50–150 nm in diameter (Fig. 1b). Western blot analysis revealed that EPC-Exos expressed exosome-specific markers CD9, Alix, and TSG101, but not Calnexin, an integral protein of the endoplasmic reticulum that is not expressed in exosomes (Fig. 1c). The exosomal miR-126 was downregulated after transfection, as revealed by qRT-PCR analysis (Fig. 1d).

### EPC-Exos accelerated osteogenesis and consolidation during DO in rats

**Fig. 2:**
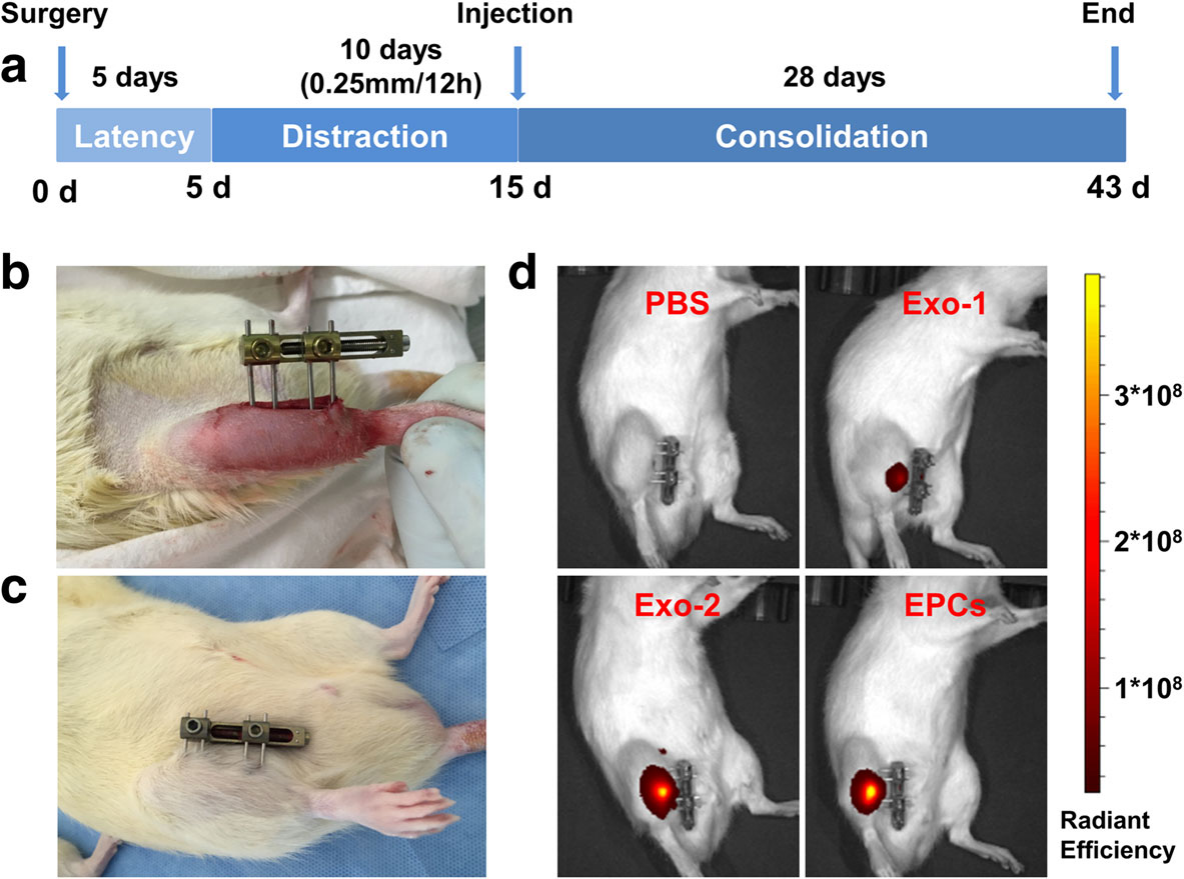
Establishment of rat tibial distraction osteogenesis model. **a** Schematic diagram illustrating the timeline of the rat tibial distraction osteogenesis procedures. **b** Representative photograph after application of external fixation. **c** Photograph at 4 weeks after consolidation. **d** In vivo imaging of Exos or EPCs stained with DiR at 2 weeks after injection. *EPCs* endothelial progenitor cells, *Exo* exosomes secreted by EPCs

No rats died or experienced evident complications during the process of DO (Fig. 2a). Representative photographs of the DO model after application of external fixation (Fig. 2b) and consolidation for 4 weeks (Fig. 2c) are shown. In vivo imaging of Exos or EPCs stained with DiR (Fig. 2d) at 2 weeks after injection indicated that both the Exos and EPCs gathered in this target region.

**Fig. 3:**
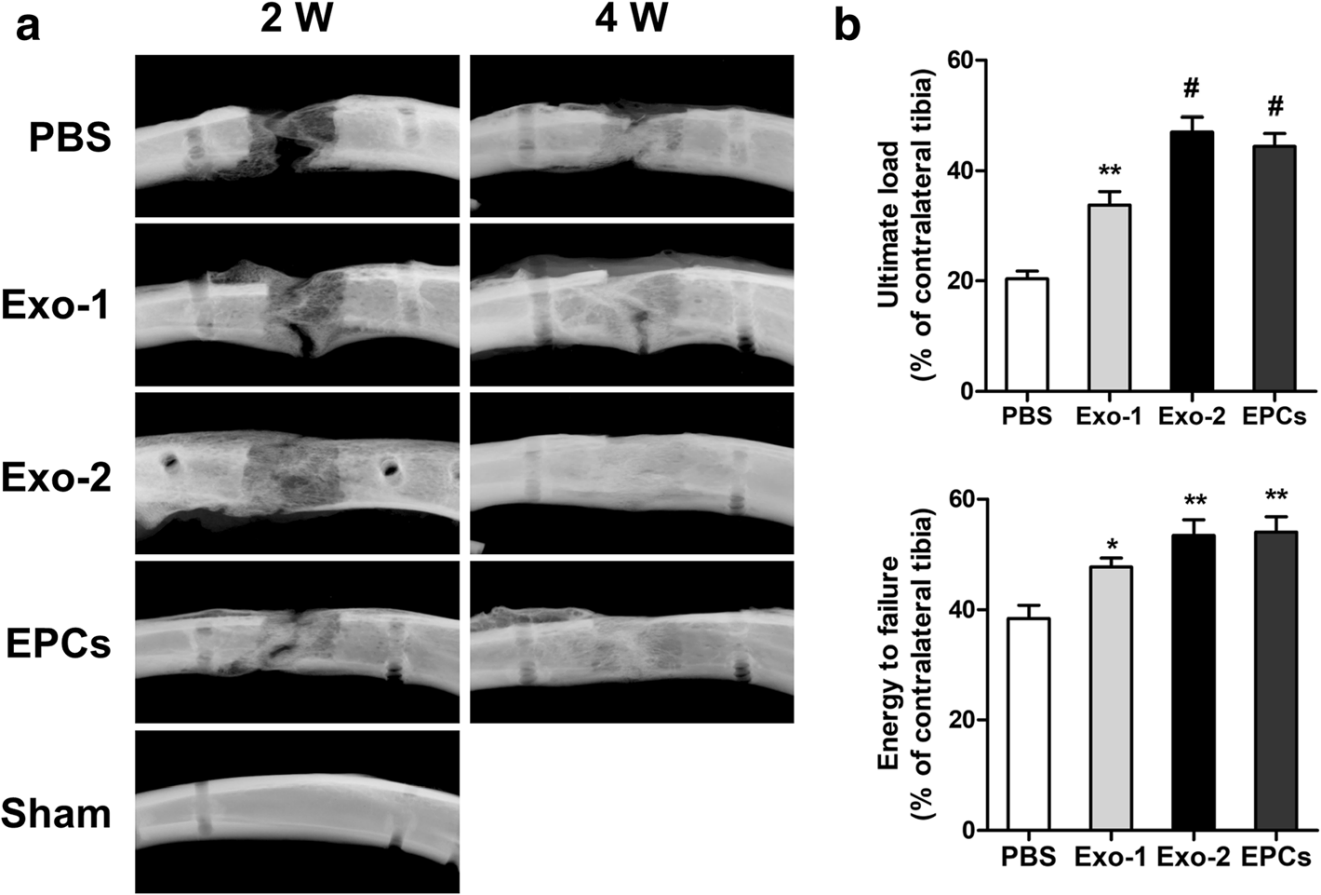
EPC-Exos accelerated bone formation and consolidation during distraction osteogenesis in rats. **a** X-ray images of the distraction regenerate at 2 and 4 weeks after distraction. **b** Mechanical tests (ultimate load and energy to failure) of the distracted tibias. The values were normalized to the corresponding contralateral normal tibias. *P < 0.05, **P < 0.01, ^#^*P* < 0.001 vs PBS group. *EPCs* endothelial progenitor cells, *Exo* exosomes secreted by EPCs, *PBS* phosphate-buffered saline

**Fig. 4:**
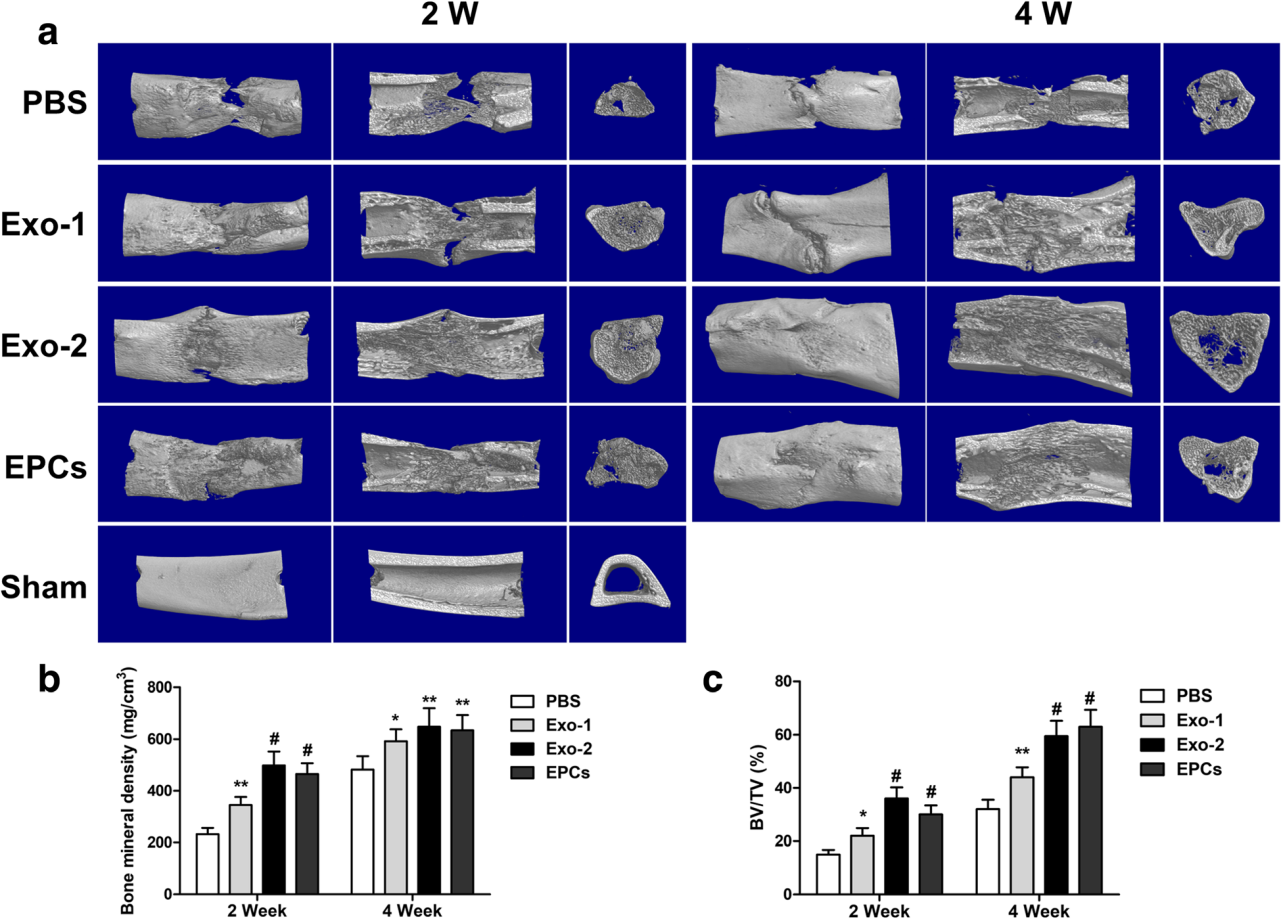
EPC-Exos accelerated bone regeneration during distraction osteogenesis in rats. **a** Representative 3D micro-CT images of the tibial distraction zone after consolidation for 2 and 4 weeks indicated more continuous callus in the Exo-2 and EPCs group. **b**, **c** Quantitative analysis of micro-CT data showed higher values of bone mineral density (BMD) and bone volume/total tissue volume (BV/TV) in the Exo-2 and EPCs group. *P < 0.05, **P < 0.01, ^#^*P* < 0.001 vs PBS group. *EPCs* endothelial progenitor cells, *Exo* exosomes secreted by EPCs, *PBS* phosphate-buffered saline

Figure 3a shows radiographs from each group. Callus formation increased with time; however, more calluses were observed in the Exo-2 and EPCs groups than in the PBS group (Fig. 3a). The results of mechanical assessments were normalized to the contralateral intact tibia. Both the values of ultimate load and energy to failure in the Exo-1, Exo-2, and EPCs groups showed significant improvement in comparison with the PBS group (*P* < 0.05) (Fig. 3b). Bone regeneration was further quantified via micro-CT at 2 and 4 weeks after DO. Representative 3D micro-CT reconstruction and 3D longitudinal and transversal profiles of the distraction gaps are shown in Fig. 4a. Both the BV/TV and BMD values were significantly higher in the Exo-1, Exo-2, and EPCs groups than in the PBS group (*P* < 0.05) (Fig. 4b, c), suggesting the beneficial effects of EPC-Exos in promoting bone regeneration.

### Histological analysis

**Fig. 5:**
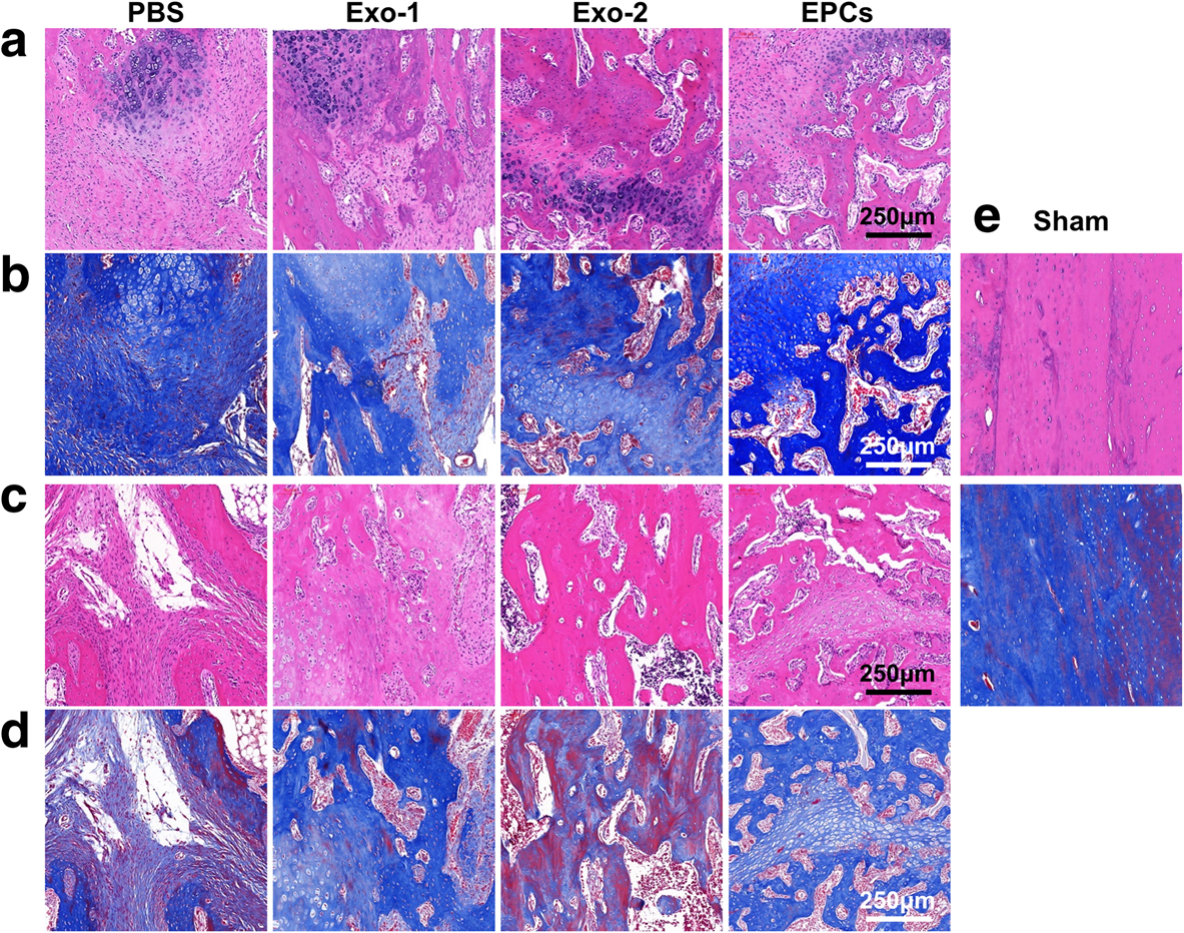
Histological analysis of the distracted regenerates from four groups after consolidation for 2 (**a**, **b**) and 4 weeks (**c**, **d**). **e** H&E staining and Masson’s staining in the sham group. The distraction gaps in all four groups mainly consisted of various amounts of trabecular bone, fibrous tissue, and cartilaginous tissue. In PBS-treated samples, the distraction gaps showed increased fibrous and cartilaginous tissues and less trabecular bone in the center zone. With treatment of Exos or EPCs, the distraction gaps showed more mature trabecular bone and less fibrous and cartilaginous tissues. *EPCs* endothelial progenitor cells, *Exo* exosomes secreted by EPCs, *PBS* phosphate-buffered saline

H&E and Masson’s trichrome staining of the distraction regenerates treated with Exo-2 and EPCs exhibited enhanced bone consolidation at 2 (Fig. 5a, b) and 4 weeks (Fig. 5c, d) after distraction in comparison with the PBS group. The distraction gaps in all four groups primarily comprised different amounts of fibrous tissue, cartilaginous tissue, and trabecular bone, parallel with the distraction forces. In PBS-treated samples, the distraction gaps contained increased fibrous and cartilaginous tissues and less trabecular bone in the central zone. Upon treatment of EPC-Exos or EPCs, the distraction gaps showed more mature trabecular bone and less fibrous or cartilaginous tissues.

### EPC-Exos stimulated angiogenesis during DO

**Fig. 6:**
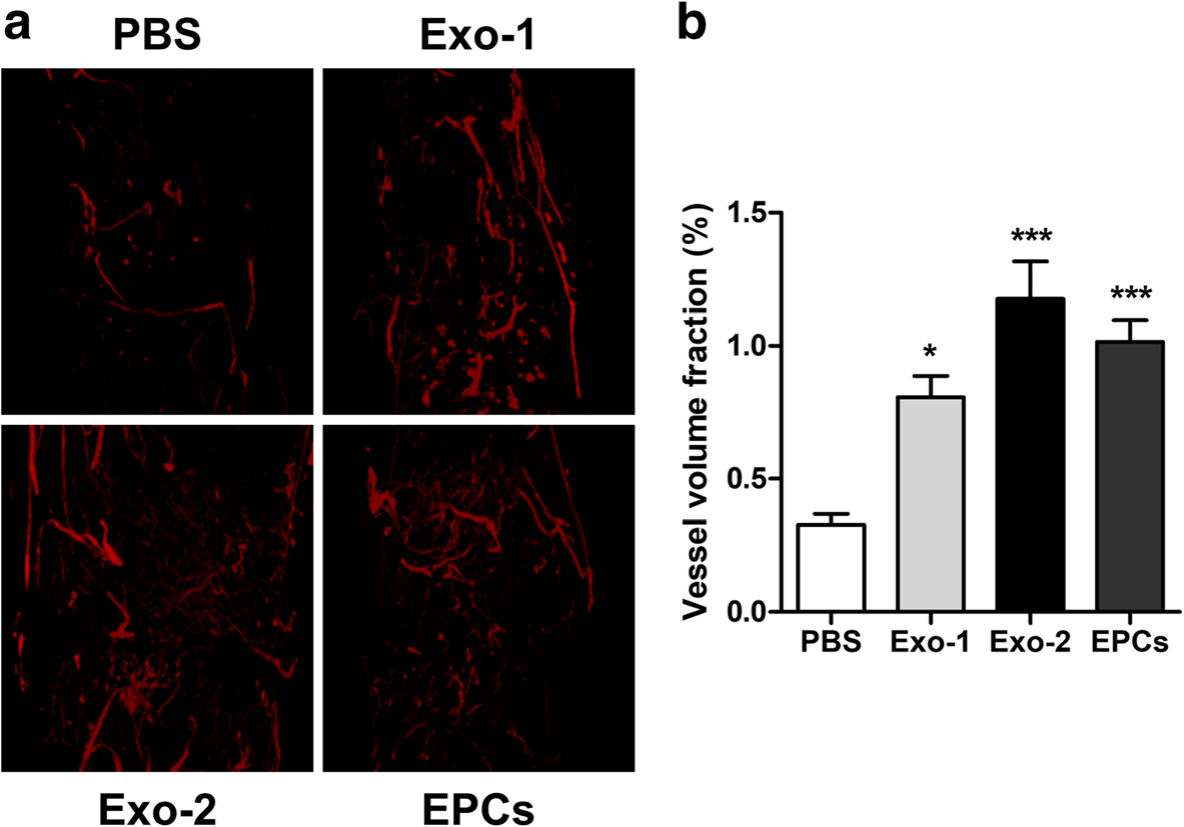
EPC-Exos enhanced the vessel density during distraction osteogenesis. **a** 3D reconstruction of the demineralized distraction regenerates perfused with Microfil at 4 weeks after consolidation. **b** Quantitative analysis of the vessel volume fractions within the distraction gaps from the four groups. *P < 0.05, ***P < 0.001 vs PBS group. *EPCs* endothelial progenitor cells, *Exo* exosomes secreted by EPCs, *PBS* phosphate-buffered saline

Since EPC-Exos reportedly promote angiogenesis in ischemic tissues, we further evaluated vessel density in the distracted gaps via Microfil perfusion [32]. Quantitative analysis revealed that vessel volume fractions in the Exo-1, Exo-2, and EPCs groups were significantly higher than those in the PBS group (*P* < 0.05) (Fig. 6a, b), indicating that EPC-Exos and EPCs effectively stimulated angiogenesis during DO.

### Immunochemical analysis

**Fig. 7:**
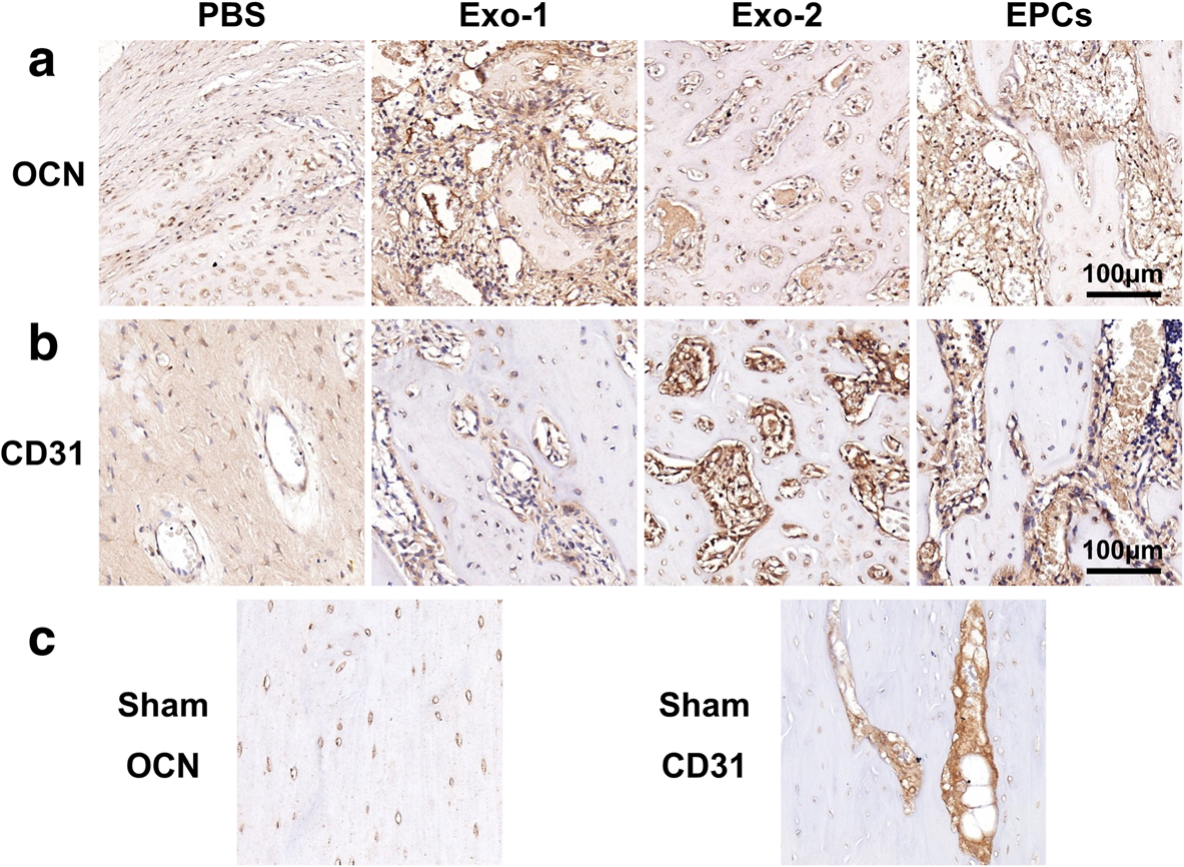
Immunohistochemical analysis of OCN (**a**) and CD31 (**b**) in the distraction zone. **c** OCN and CD31 images from the sham group. *EPCs* endothelial progenitor cells, *Exo* exosomes secreted by EPCs, *OCN* osteocalcin, *PBS* phosphate-buffered saline

Immunohistochemistry staining for OCN and CD31 was performed to detect mature osteoblasts and vessels. The results revealed more OCN-positive cells and CD31-positive vessels in the new bone zone in the Exo-1, Exo-2, and EPCs groups than in the PBS groups (Fig. 7a–c).

### EPC-Exos enhanced the proliferation, migration, and angiogenic capacity of HUVECs via exosomal miR-126

**Fig. 8:**
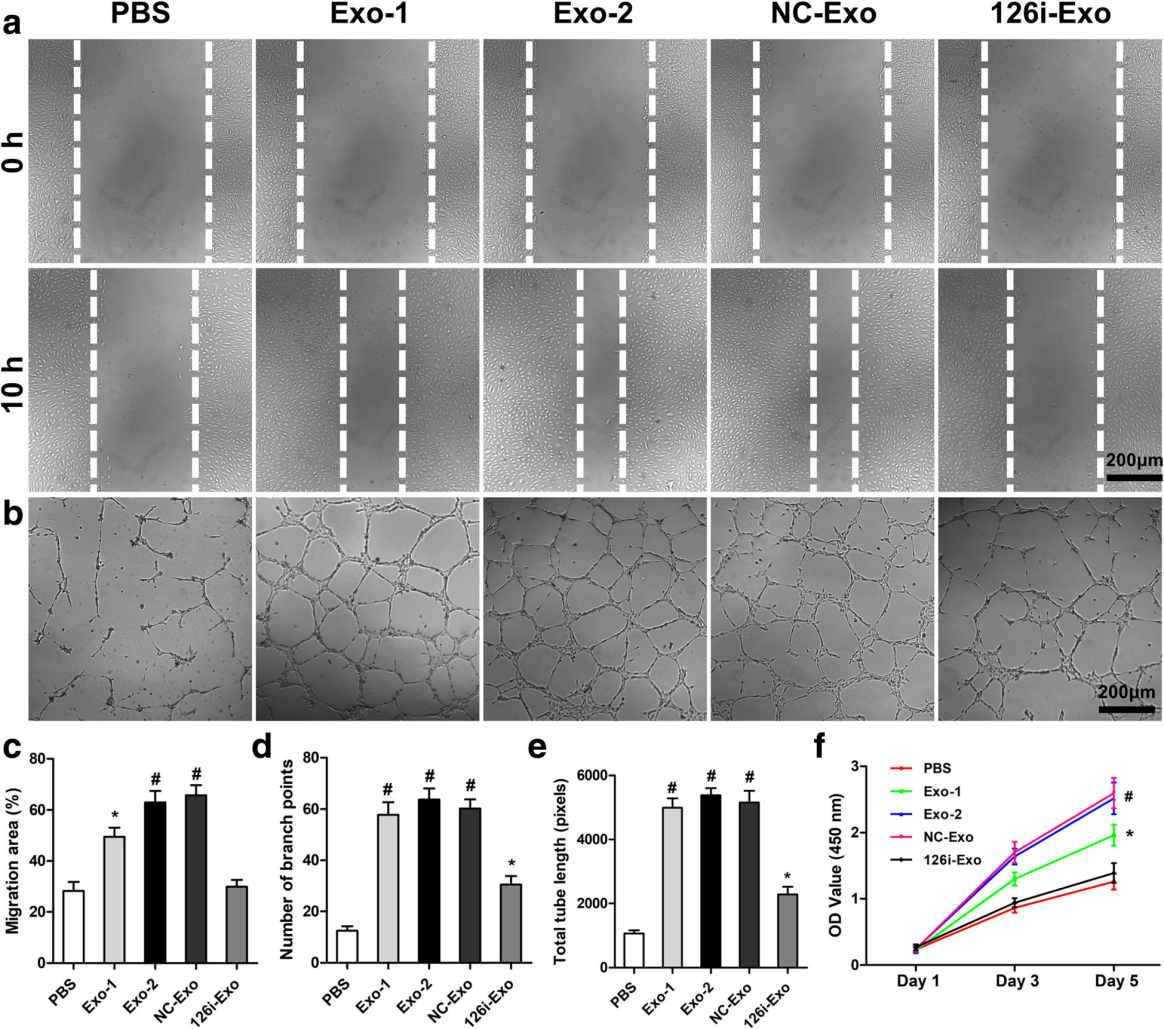
EPC-Exos enhanced the proliferation, migration, and angiogenic ability of HUVECs via miR-126. **a** Wound healing assay was used to evaluate the effect of EPC-Exos on the motility of HUVECs. **b** The tube formation assay was performed to assess the angiogenic capability of HUVECs treated with EPC-Exos or PBS. **c** Quantitative analysis of migration area indicated that EPC-Exos significantly enhanced the motility of HUVECs via miR-126. **d**, **e** Qualification of the branch points and total tube length indicated the increased angiogenic capability of HUVECs treated with EPC-Exos via miR-126. **f** The results of CCK-8 assay showed that EPC-Exos promoted proliferation of HUVECs, and a higher dose of exosomes exerted a better effect. *P < 0.05, ^#^*P* < 0.001. *HUVECs* human umbilical vein endothelial cells, *Exo* exosomes secreted by endothelial progenitor cells, *PBS* phosphate-buffered saline

**Fig. 9:**
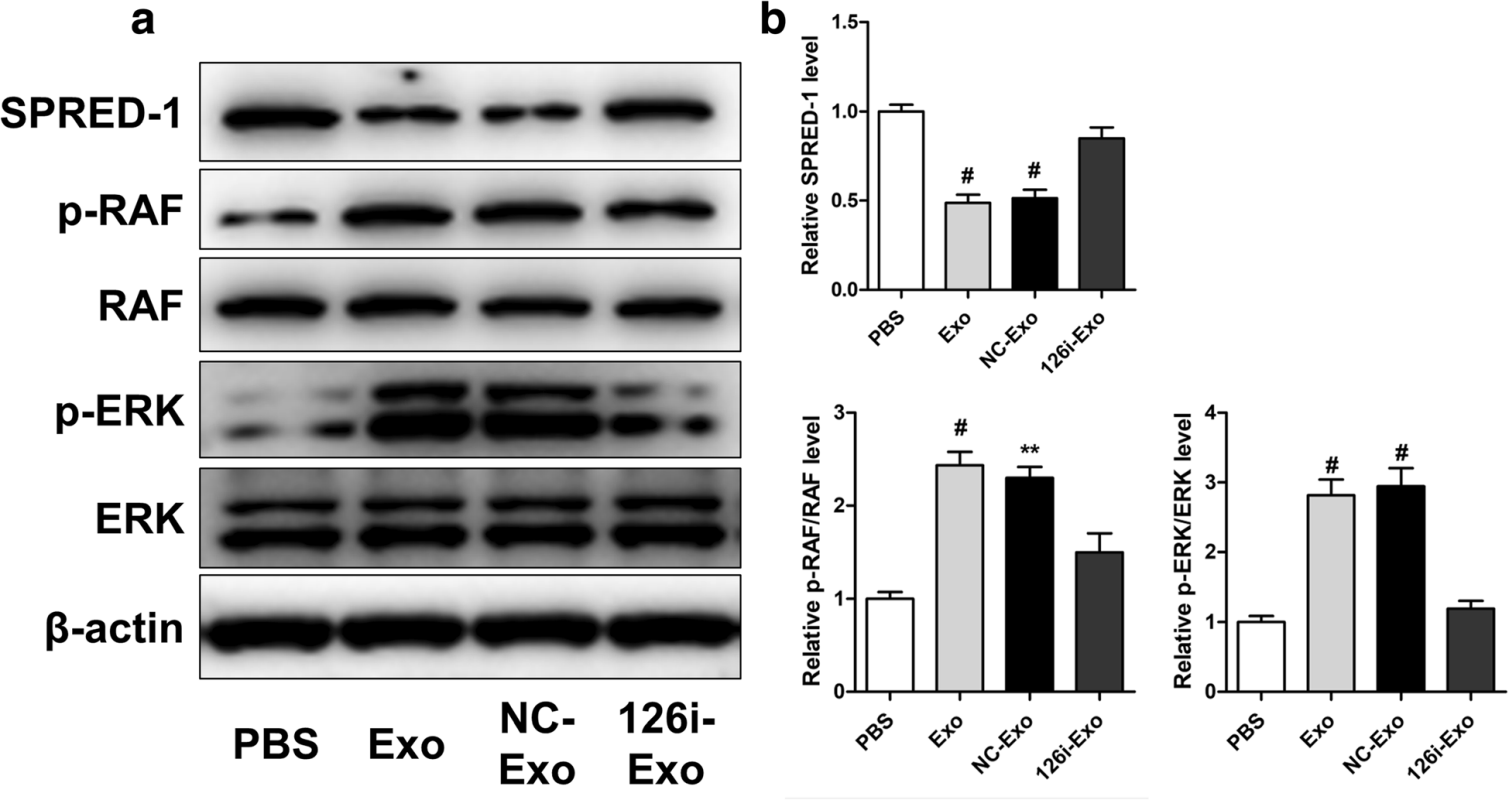
Exosomal miR-126 regulated RAF/ERK signaling through targeting SPRED-1. **a** Western blot analysis showed that EPC-Exos reduced SPRED-1 levels but induced phosphorylation of ERK and RAF. **b** Quantitative analysis of SPRED-1, p-RAF, and p-ERK. **P < 0.01, ^#^*P* < 0.001 vs PBS. *Exo* exosomes secreted by endothelial progenitor cells, *PBS* phosphate-buffered saline

Since proliferation, migration, and sprouting of endothelial cells are critical for angiogenesis, we further investigated the effects of EPC-Exos on HUVECs in vitro. Wound healing assay revealed that EPC-Exos significantly enhanced the motility of HUVECs (*P* < 0.05) (Fig. 8a, c). In addition, the total tube length and branch points were significantly higher after co-culture with EPC-Exos, as revealed by the tube formation assay at 6 h (*P* < 0.001) (Fig. 8b, d, e). Furthermore, the proliferative capacity of HUVECs treated with EPC-Exos was also significantly greater than that of the PBS group on days 3 and 5 (*P* < 0.05) (Fig. 8f). To determine the role of exosomal miR-126, EPCs were transfected with scramble miR-control and miR-126 inhibitor to prepare NC-Exos and 126i-Exos, respectively. Upon downregulation of miR-126, as revealed by qRT-PCR analysis, proliferation, migration, and tube formation were significantly decreased (*P* < 0.05) in HUVECs treated with 126i-Exos compared with those in cells incubated with EPC-Exos or NC-Exos. Western blot analysis revealed that EPC-Exos and NC-Exos but not 126i-Exos significantly downregulated SPRED1 and increased Raf and Erk1/2 phosphorylation, indicating the activation of the Erk1/2 signaling (Fig. 9a, b).

### EPC-Exos increase the expression of angiogenesis-related genes in HUVECs

**Fig. 10:**
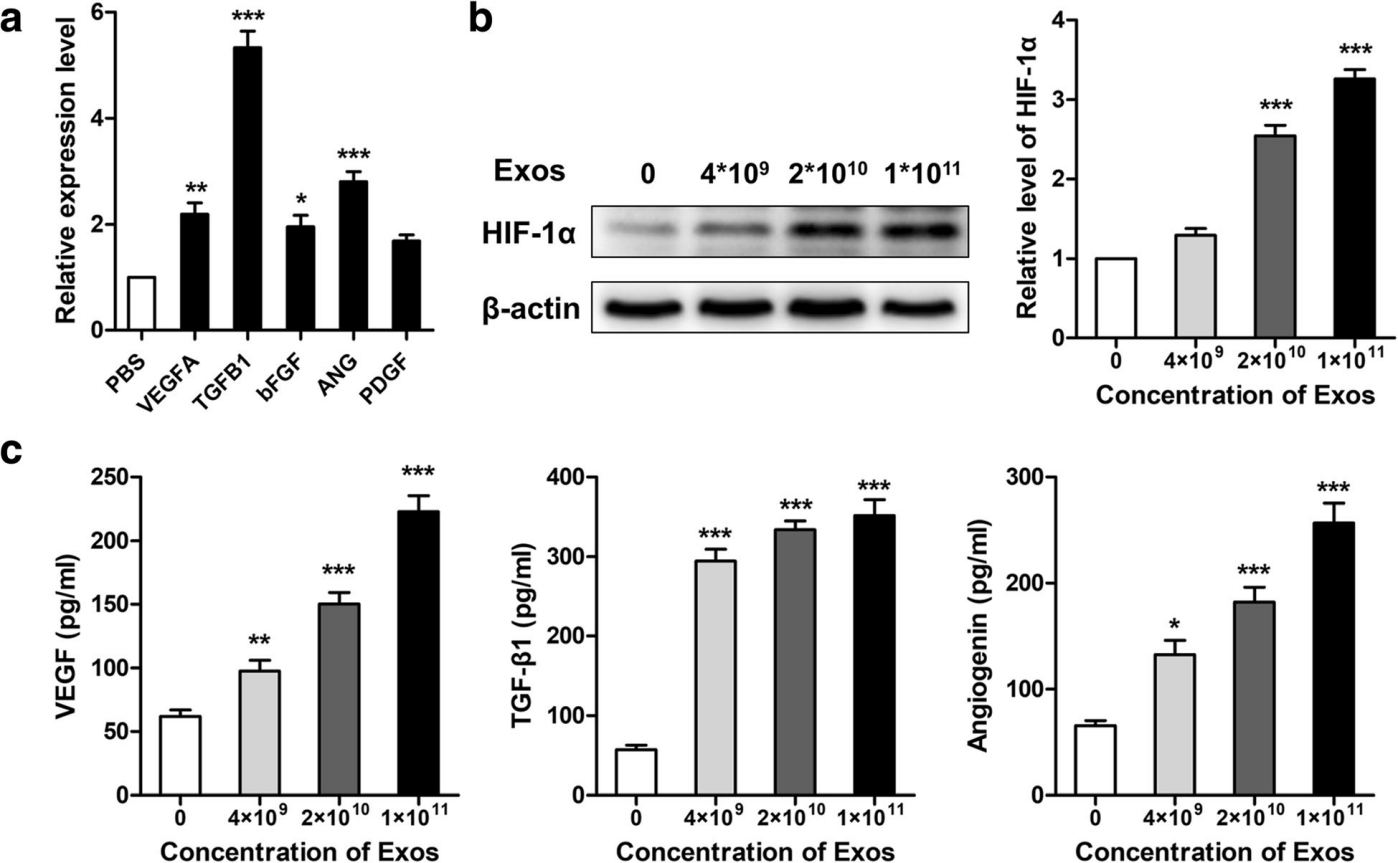
EPC-Exos enhanced the expression of angiogenesis-related genes. **a** Expression of angiogenesis-related genes in HUVECs treated with EPC-Exos (1 × 10^11^ particles/ml) or PBS using qRT-PCR. **b** Detection of HIF-1α levels in HUVECs treated with serial concentrations of EPC-Exos (0, 4 × 10^9^, 2 × 10^10^, and 1 × 10^11^ particles/ml) by western blot. **c** Concentrations of VEGF, TGF-β1, and angiogenin in the supernatants of HUVECs by ELISA. *P < 0.05, **P < 0.01, ***P < 0.001 vs PBS. *HUVECs* human umbilical vein endothelial cells, *Exo* exosomes secreted by endothelial progenitor cells, *PBS* phosphate-buffered saline

Because factors including HIF-1α, VEGF, bFGF, TGFB1, and ANG are expressed and thought to be critical in DO, their levels were detected using western blot, ELISA, and qRT-PCR. As shown in Fig. 10a, the expression levels of VEGFA, bFGF, TGFB1, and ANG in HUVECs treated with EPC-Exos were significantly higher compared to HUVECs treated with PBS (*P* < 0.05). The results of western blot and ELISA indicated that EPC-Exos could enhance the levels of HIF-1α (Fig. 10b), VEGF, TGF-β1, and ANG (Fig. 10c) in a dose-dependent manner.

## Discussion

EPCs are closely associated with the DO process. Cetrulo et al. reported that EPCs homed to the ischemic generate gaps during the distraction stage and remained in the consolidation stage in a rat mandibular DO model [33]. Lee et al. reported that DO mobilizes EPCs from the bone marrow into the peripheral blood, homing them to the distraction gaps, which may contribute to angiogenesis and bone regeneration in a rat tibial DO model [34]. Lee et al. reported the mobilization of EPCs during DO in patients undergoing limb lengthening, accompanied with elevated plasma levels of VEGF and stromal cell-derived factor 1 [35]. Furthermore, local application of stromal cell-derived factor-1 significantly enhanced bone regeneration and shortened the treatment duration of DO by mobilizing and homing EPCs in mice [36]. Collectively, it may be speculated that EPCs are critical for bone regeneration during DO.

Recent studies have supported the use of exosomes as promising alternatives for stem cell therapy [37]. EPC-Exos have been demonstrated to protect endothelial function in kidney injury, acute lung injury, and sepsis models; promote deep vein thrombosis resolution and artery re-endothelialization; and induce angiogenesis in skin wound healing [25, 38–40]. We therefore evaluated the effects of EPC-Exos on osteogenesis and consolidation in a rat tibial DO model, with EPCs as positive control. EPC-Exos and EPCs were locally injected into the distraction gap at the beginning of the consolidation phase for the following reasons. First, angiogenic factors including VEGF and bFGF were maximally upregulated during the distraction phase and were downregulated in the consolidation phase [8, 41]. Second, growth factor receptors were maximally upregulated at the beginning of the consolidation phase [42]. Consequently, transplantation of EPC-Exos and EPCs significantly accelerated callus formation and mineralization, as revealed by X-ray imaging. At 2 and 4 weeks after distraction, the quality and quantity of bone tissue in the regenerated gaps improved significantly after EPC-Exos or EPCs treatment, as revealed by histological, immunochemical, and micro-CT analyses (values of BV/TV and BMD). Furthermore, the mechanical assessment revealed improved mechanical properties (ultimate load and energy to failure) of the distraction in the EPC-Exos and EPCs group in comparison with the PBS group. Together, these results show that EPC-Exos could significantly accelerate osteogenesis and consolidation during DO in rats. It is indicated that EPC-Exos could exert the beneficial effects of EPCs whereas avoiding the possible complications of EPCs transplantation including emboli formation, immunogenicity, and malignant transformation.

Owing to the beneficial efforts of EPC-Exos in promoting bone regeneration during DO, we further explored the potential underlying mechanisms. The role of EPCs in angiogenesis is well known, and EPC-Exos have been characterized with pro-angiogenic properties derived from EPCs [25]. Microfil perfusion and immunochemical results of CD31 revealed drastically more vessels in the distraction regenerates from the EPC-Exos group. Furthermore, the CCK-8 assay, wound healing assay, and tube formation assay were performed to confirm the pro-angiogenic effects of EPC-Exos on HUVECs. These assays revealed that EPC-Exos notably increased the proliferation, migration, and angiogenic ability of HUVECs. The in vivo and in vitro results collectively supported the finding that EPC-Exos effectively stimulate angiogenesis during DO. Since angiogenesis is essential for and coupled with bone regeneration during DO, the beneficial effects of EPC-Exos on osteogenesis and consolidation during DO in this study may be at least in part attributed to enhanced angiogenesis.

We further investigated the potential mechanism underlying the promotion of angiogenesis by EPC-Exos. According to previous studies, miR-126 is predominantly enriched in EPC-Exos and critical for the functioning of EPC-Exos. We report that the pro-angiogenic effects of EPC-Exos depended on miR-126. miR-126 reportedly targets SPRED-1, which inhibits Ras/ERK signaling by blocking the Raf activation. Western blot analysis confirmed that miR-126 downregulated SPRED-1 and activated the Ras/ERK signaling pathway, concurrent with previous reports [25, 39, 40]. In addition, the present results show that EPC-Exos activated several angiogenic genes, which are expressed and critical for angiogenesis during DO [8, 41, 42]. Since exosomes contain various bioactive constituents including proteins, lipids, and RNAs, their therapeutic effects may be mediated through multiple mechanisms. Only normal EPC-Exos were investigated in this study. The limitation of this study is that the reported advantages of exosomes for drug or RNA delivery [38, 43] were not considered in the present study; hence, EPC-Exos modified to stimulate both osteogenesis and angiogenesis warrant further investigation in future studies.

## Conclusions

In summary, the present study shows that EPC-Exos promote bone regeneration during DO by stimulating angiogenesis. Furthermore, considering the promising property of EPC-Exos for harboring therapeutic molecules, these nanovesicles hold great potential to improve the quality and shorten the treatment duration of DO for treating large bone defects.

## Abbreviations

ANG: Angiogenin
bFGF: Basic fibroblast growth factor
CCK-8: Cell Counting Kit-8
DO: Distraction osteogenesis
EBM-2: Endothelial basal medium 2
EPC-Exos: Exosomes secreted by endothelial progenitor cells
EPCs: Endothelial progenitor cells
HE: Hematoxylin-eosin
HIF-1α: Hypoxia-inducible factor-1α
HUVECs: Human umbilical vein endothelial cells
PBS: Phosphate-buffered saline
qRT-PCR: Quantitative reverse-transcriptase polymerase chain reaction
SD: Standard deviation
TEM: Transmission electron microscopy
TGFB1: Transforming growth factor beta 1
TRPS: Tunable resistive pulse sensing
VEGFA: Vascular endothelial growth factor-A
